# Model-Informed Precision Dosing for Personalized Ustekinumab Treatment in Plaque Psoriasis

**DOI:** 10.3390/pharmaceutics16101295

**Published:** 2024-10-04

**Authors:** Karine Rodríguez-Fernández, Javier Zarzoso-Foj, Marina Saez-Bello, Almudena Mateu-Puchades, Antonio Martorell-Calatayud, Matilde Merino-Sanjuan, Elena Gras-Colomer, Monica Climente-Martí, Victor Mangas-Sanjuan

**Affiliations:** 1 Department of Pharmacy and Pharmaceutical Technology and Parasitology, University of Valencia, 46100 Valencia, Spain; karofer@alumni.uv.es (K.R.-F.); javier.zarzoso@uv.es (J.Z.-F.); matilde.merino@uv.es (M.M.-S.); 2 Interuniversity Research Institute for Molecular Recognition and Technological Development, Polytechnic University of Valencia-University of Valencia, 46100 Valencia, Spain; 3 Pharmacy Service, Doctor Peset University Hospital, Foundation for the Promotion of Health and Biomedical Research in the Valencian Region (FISABIO), 46017 Valencia, Spain; saez_marbel@gva.es (M.S.-B.); climente_mon@gva.es (M.C.-M.); 4 Dermatology Service, Doctor Peset University Hospital, Foundation for the Promotion of Health and Biomedical Research in the Valencian Region (FISABIO), 46017 Valencia, Spain; mateu_alm@gva.es; 5 Dermatology Service, Hospital Manises of Valencia, 46940 Valencia, Spain; martorell_antcal@gva.es; 6 Pharmacy Service, Hospital Manises of Valencia, 46940 Valencia, Spain; gras_ele@gva.es

**Keywords:** psoriasis, ustekinumab, pharmacokinetic/pharmacodynamic

## Abstract

Background/Objectives: Implementing model-informed precision dosing (MIPD) strategies guided by population pharmacokinetic/pharmacodynamic (PK/PD) models could enhance the management of inflammatory diseases such as psoriasis. However, the extent of individual experimental data gathered during MIPD significantly influences the uncertainty in estimating individual PK/PD parameters, affecting clinical dose selection decisions. Methods: This study proposes a methodology to individualize ustekinumab (UTK) dosing strategies for 23 Spanish patients with moderate to severe chronic plaque psoriasis., considering the uncertainty of individual parameters within a population PK/PD model. Results: An indirect response model from previous research was used to describe the PK/PD relationship between UTK serum concentrations and the Psoriasis Area and Severity Index (PASI) score. A maximum inhibition drug effect (I_max_) model was selected, and a first-order remission constant rate of psoriatic skin lesion (k_out_ = 0.016 d^−1^) was estimated. Conclusions: The MIPD approach predicted that 35% and 26% of the patients would need an optimized and intensified dosage regimen, respectively, compared to the regimen typically used in clinical practice. This analysis demonstrated its utility as a tool for selecting personalized UTK dosing regimens in clinical practice in order to optimize the probability of achieving targeted clinical outcomes in patients with psoriasis.

## 1. Introduction

The management of psoriasis, a chronic immune-mediated condition, has been revolutionized with the introduction of therapeutic monoclonal antibody (mAb), with ustekinumab (UTK) constituting one of the available treatment choices [1,2]. UTK targets with high specificity and affinity the shared p40 subunit of interleukin (IL)-12 and IL-23 [3,4]. Both cytokines play an important role in the immune cascade that leads to psoriasis, particularly in modulating the differentiation of naïve T-cells into Th1 and Th17 cells [5,6,7].

Under the brand name Stelara^®^ (Janssen Biotech, Inc., Horsham, PA, USA) UTK is available in solution for injection and can be administered subcutaneously. The latter formulation offers flexibility in administration, autoinjectors being the most recent [8]. The clinical development programs for UTK have been the most extensive for a biologic agent. Phase III clinical trials [9,10,11,12] for UTK in psoriasis have specified a significant decrease in Psoriasis Area and Severity Index (PASI) scores, supporting considerable improvements in the severity of the disease. Additionally, UTK has been directly compared to the tumor necrosis factor inhibitor etanercept [13], representing the first-ever head-to-head comparison of biological agents in psoriasis treatment. UTK is dosed according to body weight, as indicated in the summary of product characteristics (SmPC) [8]. For individuals weighing ≤ 100 kg and those weighing > 100 kg, a 45 and 90 mg dose is given, respectively, at weeks 0 and 4 (induction period), followed by subsequent doses every 12 weeks (the maintenance period). UTK typically reaches a steady state within approximately 28 weeks of regular dosing.

A population pharmacokinetic (PK) modeling approach was developed for UTK in clinical trial adult patients with moderate to severe plaque psoriasis [14] and psoriatic arthritis [15] and in real-world data patients [16], using a one-compartment model with first-order absorption and first-order-elimination, demonstrating a comparable PK between adult patients from clinical trials and real-world data. The same model was developed in clinical trial pediatric patients with moderate to severe plaque psoriasis [17]. In addition, an integrated population PK analysis was performed to describe the PK behavior of UTK in healthy subjects and individuals with psoriasis, psoriatic arthritis, Crohn’s disease, and ulcerative colitis [18]. The chosen structural PK model for UTK was a two-compartment open model incorporating first-order absorption and elimination kinetics. Notably, parameters such as clearance (CL) and the volume of distribution were found to increase nonlinearly with body weight. This model effectively incorporates the PK characteristics of UTK across all approved inflammatory conditions and in healthy individuals, providing a consistent framework for understanding UTK’s PK profile in diverse patient populations. The relationship between serum concentration–time data with longitudinal measures of the PASI in patients with moderate to severe plaque psoriasis was described via an indirect response model in a clinical trial and real-world patient data [16,17,19,20]. The contribution of the UTK effect to the inhibition of the formation rate of psoriatic skin lesions was described by a maximum inhibition drug effect (I_max_) model [21].

Dermatologists often follow labeled dosing recommendations for initial treatment with UTK, which may not be optimal or safe for every patient [22]. Due to variable psoriasis disease progression in individual patients and the variability in response, they perform changes in the dosing regimen of UTK during the maintenance period of treatment. These changes in routine clinical practice are called optimizations and intensifications of SmPC dosage regimens. Dose regimen optimization involves maintaining the dose but extending the dosing interval or decreasing the dose while maintaining the dosing interval. On the contrary, dose regimen intensification implies maintaining the dose but decreasing the dosing interval or increasing the dose while maintaining the dosing interval. With this approach, the risk of adverse events increases due to drug concentrations that are either supratherapeutic or subtherapeutic because of the lack of a model-informed decision-making process [23]. Additionally, the methods for individualization commonly used in standard clinical practice, such as those based on clinical response and therapeutic drug monitoring (TDM), demand strict adherence to the sampling schedule. Intervention typically occurs only after the drug has reached a steady state, and adjustments are made by comparing the patient’s exposure to a target range. If the exposure falls outside this range, the dose is adjusted under the assumption of dose–exposure proportionality at steady state or based on clinical experience [24,25,26,27].

Consequently, dermatologists require a flexible approach for psoriasis treatment that considers non-labeled dosing regimens and transitions between different therapeutic modalities of psoriasis to select the optimal treatment for each patient and to solve the cases of suboptimal response, the loss of efficacy over time, or the emergence of adverse effects. These challenges highlight the critical importance of transitioning to model-informed precision dosing (MIPD) strategies guided by population pharmacokinetic/pharmacodynamic (PK/PD) models. With MIPD, any timed sample can be utilized, allowing interventions at any point, from before the first dose to when a steady state is reached. The intervention method involves calculating a dose that achieves a predefined PK/PD target [27,28,29,30,31,32]. Implementing PK/PD models in dosing decisions for psoriasis management improves precision dosing and addresses the interindividual variability (IIV) observed in psoriasis treatment responses, enhancing therapeutic outcomes and patient safety [14,17,19,33]. Therefore, the objective of this study was to propose a methodology capable of individualizing dosing strategies of UTK in patients with moderate to severe chronic plaque psoriasis based on the uncertainty of the individual parameters of a population PK/PD model.

## 2. Materials and Methods

### 2.1. Study Design

A post-authorization, prospective, and observational clinical practice follow-up study was conducted on Spanish patients with moderate to severe chronic plaque psoriasis from Manises Hospital of Valencia. The authors affirm that all procedures undertaken in this study adhere to the ethical standards established by the pertinent national and institutional committees overseeing human experimentation. Furthermore, they ensure compliance with the Helsinki Declaration of 1975, with revisions made in 2008. The study received approval from the Ethics Committee of La Fe University and Polytechnic Hospital (protocol code VMS-UTK-2020-01 EPA-SP). All participants provided written informed consent. The enrollment period spanned from February 2021 to December 2022, encompassing individuals who had received at least one dose of UTK (Stelara^®^) and who were under treatment at the time of inclusion. The patients included in the study were receiving dosage regimens individualized (optimized/intensified or not) and authorized by the dermatologist based on their clinical response. Exclusion criteria included individuals below 18 years of age, pregnant individuals, and those with cognitive impairment. Treatment-related variables such as dosage regimen, time, and line of treatment were collected for each patient. Moreover, demographic data were extracted from the hospital’s electronic clinical records, including age, sex, weight, and height.

### 2.2. Blood Sampling and Analytical Quantification of Samples

Patients were administered UTK via subcutaneous injection in the abdomen or upper thigh. The patients received the following dosage regimen as a current treatment: 45 or 90 mg of UTK every 12, 14, and 16 weeks (q12w, q14w, and q16w), 45 mg of UTK every 18 weeks (q18w), and 90 mg of UTK every 8, 10, and 15 weeks (q8w, q10w, q15w). Blood samples for PK analysis were collected using plain red vacutainer tubes immediately before UTK administration and approximately 2, 6, 10, 12, 14, 16, and 18 weeks afterward. After centrifugation, serum samples were transferred to separate tubes and frozen at temperatures between −20 and −80 °C until processing. Serum concentrations of UTK were measured in the laboratory of Manises Hospital using an enzyme-linked immunosorbent assay (ELISA; Promonitor-UTK assay, Progenika Biopharma, Grifols^®^, Derio, Spain). The Promonitor-UTK quantifies concentrations of UTK in the range of 0.63–20 μg/mL.

### 2.3. Psoriasis Area and Severity Index Score Measurement

In the dermatology service from Manises Hospital, patients initiating UTK therapy undergo PASI assessments every 3 months during the first year of treatment. Following the initial year, PASI evaluations occur every 6 months. Baseline PASI values, recorded at the onset of UTK therapy, were extracted from patient clinical records. All available PASI scores documented in patient clinal records during the approximately 1.5-year follow-up period were collected.

### 2.4. Modeling Data Analysis

**Figure 1:**
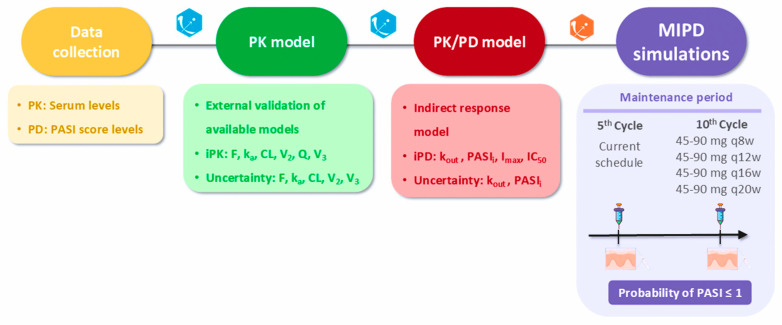
Modeling workflow. PK: pharmacokinetic; PD: pharmacodynamic; PASI: Psoriasis Area and Severity Index; iPK: individual pharmacokinetic parameter; iPD: individual pharmacodynamic parameter; F: bioavailability; k_a_: absorption rate constant; CL: clearance; Q: intercompartmental transfer clearance; V_2_: central volume of distribution; V_3_: peripheral volume of distribution; k_out_: first-order remission constant rate of psoriatic skin lesion; I_max_: maximum inhibition drug effect model; PASI_i_: estimated baseline levels of PASI response; IC_50_: concentration of the drug needed to inhibit 50% of the response; MIPD: model-informed precision dosing; q8w: once every 8 weeks, q12w: once every 12 weeks; q16w: once every 16 weeks; q20w: once every 20 weeks.

Figure 1 summarizes the modeling strategy performed. The data analysis was initiated by simulation using the population PK parameters of the 7 reference PK models published for UTK [14,15,16,17,18,34]. This aided in identifying the model that most accurately represented our data. Subsequently, we applied the chosen model along with the individual UTK serum concentrations gathered during TDM to estimate individual PK parameters.

**Figure 2:**
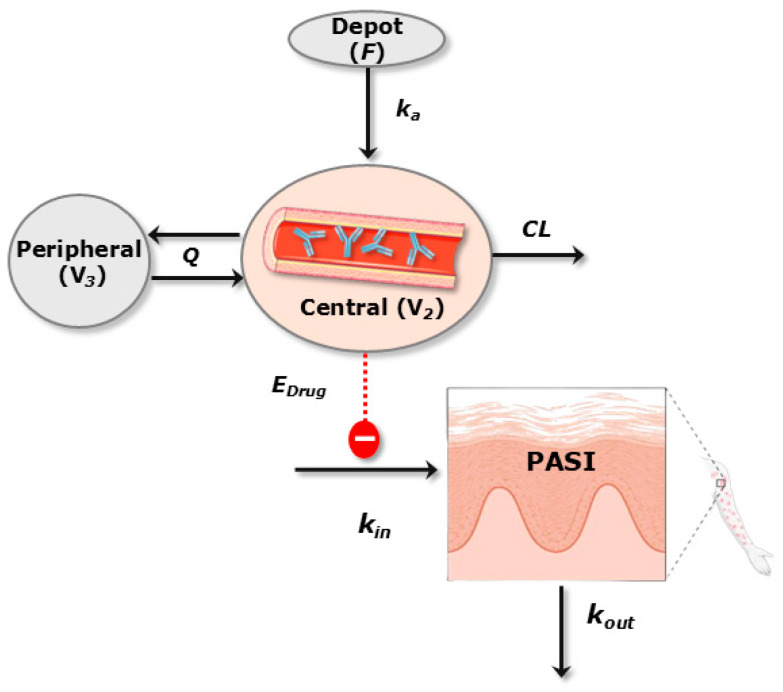
Schematic representation of the final PK/PD model. E_Drug_: effect of the drug; k_in_: zero-order progression constant rate of psoriatic skin lesion.

A previously published indirect response model with PASI synthesis inhibition [16] was considered to describe the PK/PD relationship between UTK serum concentrations and the PASI (Figure 2). The ordinary differential equations of the PK/PD model can be found in the Supplementary Materials. This model structure was also previously applied to other mAbs designed for the treatment of psoriasis [19,35,36,37,38]. Linear, I_max_, and sigmoid drug effects functions were evaluated [21].

Baseline levels of PASI response (PASI_i_) were estimated using the B2 method [39], where PASI_i_ represents the individual predicted baseline level of the PASI, PASI_i,o_ is the individual observed baseline and *η*_i,RV_ is the estimated individual random component accounting for the difference between PASI_i_ and PASI_i,o_. This variable has a mean equal to zero and, during parameter estimation, is restricted to having the same variance as the residual unexplained variability (RUV) (Equation (1)). Consequently, we assumed that the variability observed in the rest of the data also applies to the baseline data.

(1) $${PASI}_{i}={PASI}_{i,0}\cdot e^{\eta_{i,RV}}$$

The selection of the PK/PD model was based on the comparison of the minimum value of the objective function, a visual exploration of goodness-of-fit plots and the precision of model parameters stated in the standard errors. An evaluation of the selected PK/PD models was performed through simulation-based diagnostics prediction-corrected visual predictive checks (pcVPCs) [40,41]. All data analyses were performed based on the population approach with the software Monolix 2024R1 (Lixoft SAS, a Simulations Plus company, Lancaster, CA, USA) [42]. For graphical and statistical analysis, R software (http://cran.r-project.org, version 4.4.1, accessed on 30 September 2024) was employed [43,44].

### 2.5. Individual Dosing Regimen Strategy

To explore the performance of the final PK/PD model and its impact on clinical practice, the optimal MIPD regimen in each patient was determined, for which the PK and PD individual parameters and their uncertainties were considered. The individual conditional distributions of PK/PD parameters were estimated in Monolix, which represented the PK and PD individual parameter estimates and their corresponding uncertainty [45]. Uncertainty refers to the degree of accuracy with which PK and PD individual parameters can be determined based on the observed data and covariate value for that individual, considering that the individual belongs to the population for which the typical parameter value (fixed effects) and the variability (standard deviation of the random effects) were previously estimated [45,46]. 

The determination of individual conditional distributions ($p(\psi_{i}|y_{i})$, with $\psi_{i}$ being the individual parameters for individual *i* and $y_{i}$ the data observations for individual *i*), was performed with a Markov Chain Monte Carlo (MCMC) procedure called the Metropolis–Hastings algorithm that samples parameter values from these distributions, using the following expression:

(2) $$p(\psi_{i}|y_{i})=\frac{p\left(y_{i}|\psi_{i}\right)\cdot \;p(\psi_{i})}{p(y_{i})}$$

where $p\left(y_{i}|\psi_{i}\right)$ is the conditional density function of the data when knowing the individual parameter values, $p(\psi_{i}$) is the density function for the individual parameters and $p(y_{i})$ is a constant that represents the likelihood.

With individual conditional distributions, one hundred clones per patient were generated and were imputed to Simulx 2024R1 (Lixoft SAS, a Simulations Plus company) [47] to predict through stochastic simulations, and the PASI levels expected at the 5th and 10th cycle (maintenance period) of treatment with UTK were as follows:

- 5th cycle: individual simulations of dosing regimens were generated considering 5 cycles of UTK (steady-state conditions) administration with the administered dosage regimen of each patient.
- 10th cycle: following the 5th cycle, we simulated the combination of alternative dose levels (45 and 90 mg) with different posology (q8w, q12w, q16w, and q20w) for each patient during 5 more cycles of treatment with UTK.

For each patient, the probabilities of achieving the response target (a PASI score ≤ 1) with each simulated dose regimen were calculated in cycle 5 and 10 using the following formula:

(3) $$Probability=\frac{n_{PASI}}{T_{PASI}}\times 100$$

where $n_{PASI}$ represent the amount of simulated PASI score values that reach the response target (a PASI score ≤ 1), and $T_{PASI}$ represent the total amount of simulated PASI score values (100 clones). In the 10th cycle, the regimen with which the patient achieved a probability ≥ 90% (i.e., ≥90 virtual patients) was selected as the final dosage regimen. In case the patient achieved the response target with more than one dosage regimen, the optimized dosing regimen was selected as the final regimen, which represented a maintenance of the dose over a wider dosing interval or a decrease in the dose within the same dosing interval. The dosage regimen selected in the 10th cycle was compared with the current dosage regimen from the clinical practice of each patient to determine if the patients continued with their current regimen or required a change (optimization or intensification) in the dosage regimen.

## 3. Results

### 3.1. Study Population

The modeling dataset consisted of 23 patients including 75 observations in serum samples (PK), a baseline PASI (${PASI}_{i,0})$ for each patient, and 117 individual PASI (PD). Table 1 summarizes the characteristics of the study, including demographic data, comorbidities, TDM data, and treatment characteristics that describe the current dosage regimen from the clinical practice of the subjects enrolled.

### 3.2. Population PK Model

**Figure 3:**
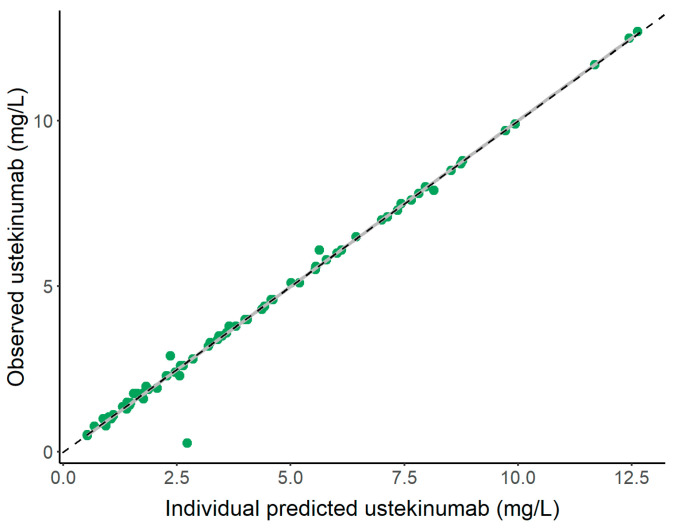
Individual predicted vs. the observed concentrations of UTK in patients with chronic plaque psoriasis.

A two-compartment model with first-order absorption and linear disposition processes, previously published by Shao et al. [18], was considered as the population PK model (Figure 2). Then, the statistical significance of covariates included in the reference model was tested with the available information and patient population collected. Only the effect of body weight was maintained on CL, intercompartmental transfer clearance (Q), the central volume of distribution (V_2_), and the peripheral volume of distribution (V_3_). Therefore, body weight was the only covariate that influenced the determination of individual PK parameter values. The results from the model evaluation exercise indicate that the model could capture the individual PK profiles with adequate accuracy since most of the observations were aligned to the identity line (Figure 3). The pcVPC of the PK model is shown in Supplementary Figure S1. 

### 3.3. Population PK/PD Model

**Figure 4:**
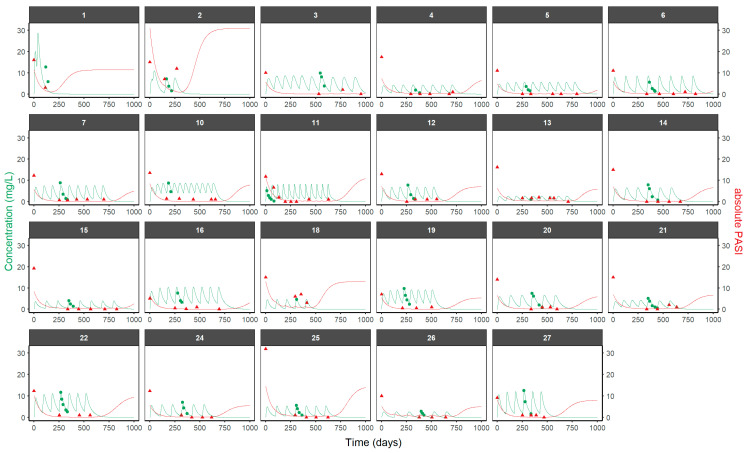
Individual predicted and observed UTK serum concentrations (green) and PASI (red) after UTK administration in patients with chronic plaque psoriasis. The line represents the individual prediction, and the green and red dots represent the UTK and PASI observations, respectively.

The relationship between UTK concentration and PASI observations was described by an indirect response [48] in which UTK inhibited the zero-order progression constant rate of psoriatic skin lesion (k_in_) through an I_max_ model. The first-order remission constant rate of psoriatic skin lesion (k_out_) and the baseline PASI levels using the B2 method were estimated. The concentration of the drug needed to inhibit 50% of the response (IC_50_) was fixed to a published value [16]. Longitudinal individual PK and PD profiles are shown in Figure 4. Overall, the PK/PD model can characterize the longitudinal behavior of the PASI at the individual level, suggesting that the current framework considers properly the combined drug effect and PASI turnover. The pcVPC of the PK/PD model is shown in Supplementary Figure S2. Parameter estimates of the final population PK/PD model are summarized in Table 2. The mean and standard deviation of individual PK/PD parameters are summarized in Supplementary Tables S1 and S2, respectively.

### 3.4. Individual Dosing Regimen Evaluation

**Figure 5:**
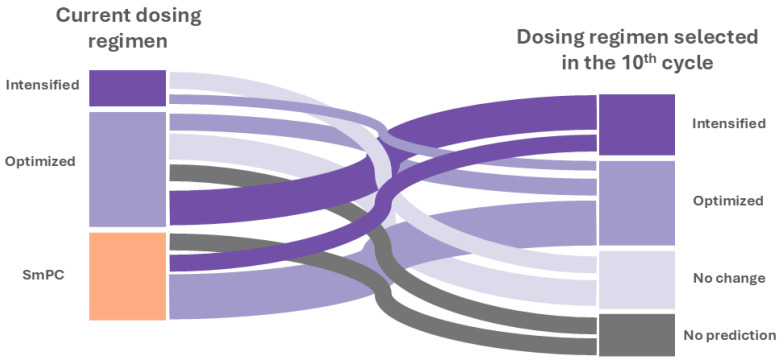
Sankey diagram to indicate the main flows of changes in the individual dose regimen from the current dosage regimen of clinical practice to the predicted dosage regimen in the maintenance period of treatment with UTK (cycle 10). SmPC: summary of product characteristics.

Probabilities across the cycles of treatment evaluated per patient are represented in Supplementary Figure S3. The MIPD strategy predicted that 8/23 (35%) and 6/23 (26%) of patients would require an optimized and intensified dosage regimen, respectively, compared with the current regimen received in clinical practice for the maintenance period. A change to the approved regimen of 45 mg q12w is proposed in 3/23 (13%), representing 38% (3/8) of the total optimized patients. Also, in the optimized patients, non-labeled dosing regimens of 45 mg q16w and 45 mg q8w are suggested in 2/8 (24%) and 3/8 (38%) of patients, respectively. Regarding the patients with intensified dosage regimens, a change to 90 mg q8w (3/6) and 45 mg q8w (3/6), respectively, is recommended. No change in the dosing regimen was predicted in 5/23 (22%) patients. This MIPD strategy additionally allowed us to identify that 4/23 (17%) patients would not achieve the efficacy endpoint selected (a 90% probability of a PASI ≤ 1), contributing to the early identification of patients with therapeutic failure (Figure 5).

**Figure 6:**
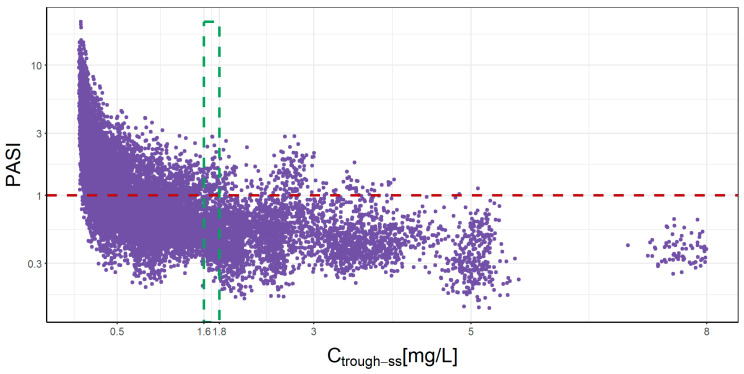
Simulated absolute PASI and trough concentration for each patient after 10 cycles of UTK administration using labeled and non-labeled dosing schemes. C_trough-ss_: trough concentration at steady state.

PK and PD simulations with the current dosage regimen from the clinical practice of each subject and the individual optimal dosing regimen established in the 10th cycle are depicted in Supplementary Figure S4. Figure 6 represents the relationship between the absolute PASI and a trough concentration at a steady state (C_trough-ss_) for each patient, with all dosage regimens tested. Overall, the results suggest a non-linear relationship, indicating that the range of concentration 1.6–1.8 mg/L allows to achieve 90% of patients with a PASI ≤ 1.

## 4. Discussion

In this study, we developed a procedure capable of individualizing dosing strategies of UTK in clinical practice Spanish patients with moderate to severe chronic plaque psoriasis based on the uncertainty of the individual parameter estimates of a population PK/PD model. Additionally, the inclusion of the uncertainty during the simulation step for a personalized dosing regimen selection facilitates the evaluation of the degree of certainty in model-informed predictions in the clinical setting, providing a probabilistic framework for the MIPD of UTK. 

Of the total number of patients who need a dosage regimen modification, 35% would be oriented towards an optimized dosage regimen. In 22% of patients, this optimization is proposed to be performed using a non-labeled dosage regimen. This represents a reduction of one dose per year, meaning that patients, instead of receiving five doses of UTK in the first year of treatment with SmPC dosing regimens and four from the second year onwards, would receive four doses and three doses, respectively. According to the database of health information on medicines and parapharmacy products [49] the price of Stelara^®^ 45 mg solution for injection in a pre-filled syringe is EUR 2915.4. Assuming the reduction in dosage, this will entail an annual cost per patient of EUR 11,661.6, which will represent a saving of 25%. In addition to fewer expenses related to the purchase of medication, with the lower number of administrations, there is less risk of an occurrence of injection site reactions due to SmPC. The previous fact includes patients who are to receive 45 mg q16w and also those who are to receive 45 mg q8w because the latter take much more intensive dosage regimens during clinical practice, for example, 90 mg q10w.

From our pool of patients, an intensification of the dosage regimen in comparison with the one received in clinical practice is proposed in 26% of the patients. In these patients, individual CL values higher than the typical value are observed; hence, this type of patient will require higher levels of UTK to achieve a successful therapeutic response. It should be taken into consideration that after performing our strategy for MIPD in UTK, dosage regimen optimization is recommended for most of the patients. As a result, an improvement in patient well-being and health system management can be achieved because, in the end, the patients would always be in an optimal therapeutic response range.

The MIPD strategy did not allow us to reach the efficacy endpoint (a 90% probability of a PASI ≤ 1) in 17% of patients. This strategy represents an advantage in identifying non-responding patients at the early stages of treatment, improving the effectiveness in the management of psoriasis for those patients, and avoiding the cost of treating these patients during successive cycles. A more in-depth analysis of these patients reveals CL or k_out_ values higher and lower, respectively, than the population average (Supplementary Figure S3), which could serve as a threshold to characterize patients with a low probability of optimal response to UTK.

For the estimation of individual PK/PD parameters, all the PASI and UTK concentration values available at the time of recruitment were used. To implement the proposed MIPD strategy, an adaptive procedure might be implemented. This procedure would involve re-estimating the individual parameters as additional data points become available to ensure the model remains accurate and reflective of the patient’s evolving condition. On the other hand, this analysis confirms the need to collect PASI samples from patients during the first weeks of treatment (the induction phase), which enhances the characterization of PK and PD processes and increases the number of observations per patient, leading to a precise and accurate estimation of their individual PD parameters. The non-linear relationship between the PASI score and trough concentration levels of UTK (Figure 6) demonstrates both the impact of the PK and PD parameters to guarantee satisfactory treatment efficacy. Therefore, although it is possible to establish global exposure values for a PASI value, the individualization of treatment through this MIPD strategy allows for the achievement of a greater number of patients with effective therapeutic regimens, reducing therapeutic failure only for non-responding patients. In our study, a C_trough-ss_ range of 1.6–1.8 mg/L was able to help 90% of patients achieve a PASI ≤ 1. Even though the C_trough-ss_ range obtained in a previous population PK/PD study [17] was 0.0914–0.978 mg/L, it should be noted that this range is representative only for patients weighing 60 to 100 kg with a dosing regimen of 45 mg q12w. Correspondingly, it was generated from the simulated data of patients who were exclusively starting treatment with UTK in pivotal phase III clinical trials, with the efficacy objective of PASI 75 in week 12. However, the range of our study represents a more demanding PD endpoint (PASI ≤ 1 or PASI 99), and it is representative of patients with no body weight limit (92 ± 18.4 [70–135] kg) receiving eight dosage regimens used in clinical practice (45 or 90 mg q8, 12, 16 and 20w) under steady-state UTK therapy conditions. Furthermore, this range provides target C_trough-ss_ values that are associated with an optimal response in line with current clinical guideline recommendations (PASI ≤ 1) intended to maximize both clinical outcomes and the quality of life of patients with psoriasis [50,51].

Our work is based on previously published PK /PD models, thus recycling data and using previously developed knowledge, but in addition to our model, we have taken a step forward in the characterization of the relationship between UTK concentrations and response through the PASI observed in real-world clinical data. But also, this study is an example of individual-level precision dosing, which involves a more comprehensive and effective use of precision dosing strategies by recognizing, characterizing, and quantifying the various sources of variability in drug response through PK/PD modeling [23,33]. Such studies are particularly valuable for drugs like UTK that are used to treat heterogeneous conditions like psoriasis, where response variability is significant. While improving our understanding of PK/PD relationships within specific patient subgroups during clinical trials can enhance the identification of precise dosing targets, real-world patients remain significantly more diverse than those in controlled trial settings. Therefore, robust post-marketing surveillance is essential to explore unique precision dosing targets [52]. Advances in data collection and analysis methods from real-world patients offer opportunities to identify new precision dosing algorithms and refine existing ones established in clinical trials. These methods enable the continuous updating and extension of dosing strategies to better meet the individualized needs of patients [33,53]. Thus, our strategy based on MIPD in real-world clinical data could be the first attempt to ensure the most precise individual dosing of patients with plaque psoriasis treated with UTK.

Similar to any real-world study cohort, notable challenges involve the lack of patients (a small patient sample size), a few points available per patient, and therefore the presence of missing data. Due to the study design conditions, many PK/PD observations were taken very spaced out in time, without a continuous characterization (sparse data). For this reason, some unquantified changes in the physiology or the progression of the psoriasis disease could directly impact the obtained results. In the future, similar models could be implemented into a conditional distribution dashboard system [54,55]. This would allow for real-time predictions of treatment response, aiding in informed decisions regarding dosing adjustments and treatment transitions for UTK. In this way, as suggested by Mould and Upton in their recently published review [56], options for alternative dosing strategies based on MIPD and/or TDM could be included in the SmPC of UTK.

## 5. Conclusions

In conclusion, this study proposes a methodology to individualize UTK dosing strategies considering the uncertainty of individual parameters within a population PK/PD model to optimize the probability of achieving targeted clinical outcomes in patients with moderate to severe chronic plaque psoriasis. This represents an initial step towards performing MIPD for biologics that act on the interleukin pathways of psoriasis, which has been explored very little. Future studies should implement our proposal in a greater cohort of real-world patients.

## Figures and Tables

**Table 1:** Summary of patients’ characteristics and PK/PD experimental data collected during TDM.

		Mean ± SD		Range		n (%)	
Demographic data							
Age (years)		62 ± 8.19		45–76			
Body weight (kg)		92 ± 18.4		70–135			
Height (m)		1.67 ± 0.06		1.54–1.83			
BMI (kg/m^2^)		32.3 ± 6.8		24–50.19			
Gender (male)						14 (64)	
Treatment period (years)		5.43 ± 3.3		0.304–11.4			
Biological “naive”						20 (87)	
Comorbidities							
AHT						2 (9)	
Dyslipidemia						2 (9)	
Diabetes						1 (4)	
Obesity						4 (16)	
Psoriatic arthropathy						2 (9)	
Non-alcoholic fatty liver						2 (9)	
Anxious–depressive disorder						2 (9)	
Others						4 (16)	
TDM data							
Total of patients						23	
UTK concentration (mg/L)		4.1 ± 3.06		0.27–12.7			
Total of UTK concentrations						75	
PASI (no units)		1.096 ± 2.13		0–12			
Total of PASIs						117	
${PASI}_{i,0}$		14.4 ± 6.23		5–31.9			
Current treatment characteristics							
SmPC		Optimized		Intensified		Summary	
45 mg q12w	1	45 mg q14w	1	90 mg q10w	1	SmPC	9 (40%)
90 mg q12w	8	45 mg q16w	3	90 mg q8w	2	Optimized	11 (48%)
		45 mg q18w	1			Intensified	3 (12%)
		90 mg q14w	1				
		90 mg q15w	2				
		90 mg q16w	3				

SD: standard deviation; BMI: body mass index; AHT: arterial hypertension; TDM: therapeutic drug monitoring; UTK: ustekinumab; PASI: Psoriasis Area and Severity Index; PASI_i,o_: individual observed baseline; SmPC: summary of product characteristics; q8w: once every 8 weeks; q10w: once every 10 weeks; q12w: once every 12 weeks; q14w: once every 14 weeks; q15w: once every 15 weeks; q16w: once every 16 weeks; q18w: once every 18 weeks.

**Table 2:** Population PK/PD estimates after administration of UTK in patients with psoriasis.

Parameter (Units)	Value	RSE (%)
Fixed effect		
k_out_ (d^−1^)	0.016	22
I_max_	0.97	0.7
IC_50_ (mg/L)	0.07 FIX	
Inter-individual variability		
k_out_ (%)	55.87	38.9
Residual unexplained variability		
Error (%)	0.86	9.95

k_out_: first-order remission constant rate of psoriatic skin lesion; I_max_: maximum inhibition drug effect; IC_50_: concentration of the drug needed to inhibit 50% of the response.

## Data Availability

The data presented in this study are available on request from the corresponding author due to ethical and legal restrictions.

## Supplementary Materials

The following supporting information can be downloaded at: https://www.mdpi.com/article/10.3390/pharmaceutics16101295/s1, Ordinary differential equations for the PK/PD model of UTK and PASI; Figure S1: Prediction-corrected visual predictive check obtained from one thousand simulated studies using the selected population PK model; Figure S2: Prediction-corrected visual predictive check obtained from one thousand simulated studies using the selected population PK/PD model; Table S1: Mean of the individual PK/PD parameters drawn from the conditional distribution task in Monolix; Table S2: Standard deviation of the individual PK/PD parameters drawn from the conditional distribution task in Monolix; Figure S3: Bar plot of 100 simulated absolute PASI for each patient after UTK administration at cycles 5 and 10, using the individual parameters from the final population PK/PD model and their uncertainties; Figure S4: PK and PD simulations with the current dosage regimen from clinical practice and the individual optimal dosing regimen established after simulations in 10th cycle for each patient.

## Abbreviations

AHT	Arterial hypertension
BMI	Body mass index
C_trough-ss_	Trough concentration at steady state
CL	Clearance
E_Drug_	Effect of the drug
F	Bioavailability
IC_50_	Concentration of the drug needed to inhibit 50% of the response
I_max_	Maximum inhibition drug effect
IIV	Interindividual variability
IL	Interleukin
k_a_	Absorption rate constant
k_in_	Zero-order progression constant rate of psoriatic skin lesion
k_out_	First-order remission constant rate of psoriatic skin lesion
mAb	monoclonal antibody
MCMC	Markov Chain Monte Carlo
MIPD	Model-informed precision dosing
$n_{PASI}$	Amount of simulated PASI score values that reach the response target (PASI score ≤ 1)
*η*_i,RV_	Estimated individual random component accounting for the difference between PASI_i_ and PASI_i,o_
PASI	Psoriasis Area and Severity Index
PASI_i_	Estimated baseline levels of PASI response
PASI_i,o_	Individual observed baseline levels of PASI response
pcVPC	Prediction-corrected visual predictive check
PD	Pharmacodynamic
PK	Pharmacokinetic
PK/PD	Pharmacokinetic/pharmacodynamic
Q	Intercompartmental transfer clearance
q8w	Once every 8 weeks
q10w	Once every 10 weeks
q12w	Once every 12 weeks
q14w	Once every 14 weeks
q15w	Once every 15 weeks
q16w	Once every 16 weeks
q18w	Once every 18 weeks
q20w	Once every 20 weeks
RUV	Residual unexplained variability
SD	Standard deviation
SmPC	Summary of product characteristics
TDM	Therapeutic drug monitoring
$T_{PASI}$	Total amount of simulated PASI score values (100 clones)
UTK	Ustekinumab
V_2_	Central volume of distribution
V_3_	Peripheral volume of distribution
