# Supplementary material for: Model-Informed Precision Dosing for Personalized Ustekinumab Treatment in Plaque Psoriasis
Source: Pharmaceutics. 2024 Oct 4;16(10):1295. doi: 10.3390/pharmaceutics16101295 (PMC11510411; doi:10.3390/pharmaceutics16101295)
Supplement: Supplementary file 1 [file pharmaceutics-16-01295-s001.zip › pharmaceutics-3216113-supplementary.pdf]

Supplementary materials

Model-Informed Precision Dosing for Personalized Ustekinumab Treatment in Plaque Psoriasis

Karine Rodriguez-Fernandez [1,2,†], Javier Zarzoso-Foj [1,2,†], Marina Saez-Bello [3], Almudena Mateu-Puchades [4], Antonio Martorell-Calatayud [5], Matilde Merino-Sanjuan [1,2], Elena Gras-Colomer [6], Monica Climente-Marti [3] and Victor Mangas-Sanjuan [1,2,*]

[1] Department of Pharmacy and Pharmaceutical Technology and Parasitology, University of Valencia. Valencia, Spain; karofer@alumni.uv.es (K.R.-F.); javier.zarzoso@uv.es (J.Z.-F.); matilde.merino@uv.es (M.M.-S.)
[2] Interuniversity Research Institute for Molecular Recognition and Technological Development, Polytechnic University of Valencia-University of Valencia. Valencia, Spain
[3] Pharmacy Service, Doctor Peset University Hospital, Valencia. Foundation for the Promotion of Health and Biomedical Research in the Valencian Region (FISABIO), Valencia, Spain; saez_marbel@gva.es (M.S.-B.); climente_mon@gva.es (M.C.-M.)
[4] Dermatology Service, Doctor Peset University Hospital, Valencia. Foundation for the Promotion of Health and Biomedical Research in the Valencian Region (FISABIO), Valencia, Spain; mateu_alm@gva.es
[5] Dermatology Service, Hospital Manises of Valencia, Spain; martorell_antcal@gva.es
[6] Pharmacy Service, Hospital Manises of Valencia, Spain; gras_ele@gva.es
* Correspondence: victor.mangas@uv.es; Tel.: +34963543351
† These authors contributed equally to this work.

Ordinary differential equations for the PK/PD model of UTK and PASI

$$\frac{dA}{dt} = -\boldsymbol{k_a} \cdot \boldsymbol{A} \qquad \text{Equation S1}$$

$$\frac{dC_C}{dt} = \boldsymbol{k_a} \cdot \boldsymbol{A} - \boldsymbol{CL} \cdot \boldsymbol{C_C} + \boldsymbol{Q} \cdot (\boldsymbol{C_p} - \boldsymbol{C_c}) \qquad \text{Equation S2}$$

$$\frac{dC_P}{dt} = -\boldsymbol{Q} \cdot (\boldsymbol{C_p} - \boldsymbol{C_c}) \qquad \text{Equation S3}$$

$$\frac{dPASI}{dt} = \boldsymbol{k_{in}} \cdot \left(\boldsymbol{1} - \frac{I_{max} \cdot C_C}{(IC_{50} + C_C)}\right) - \boldsymbol{k_{out}} \cdot \boldsymbol{PASI} \qquad \text{Equation S4}$$

Where k_a: absorption rate constant; A: amount of UTK in the depot compartment.; CL: clearance; C_c: concentration of UTK in the central compartment; Q: intercompartmental transfer clearance; C_p: concentration of UTK in the peripheral compartment; k_{in}: zero-order progression constant rate of psoriatic skin lesion; k_{out}: first-order remission constant rate of psoriatic skin lesion; I_{max}: maximum inhibition drug effect model; IC_{50}: concentration of UTK needed to inhibit 50% of the response; PASI: Psoriasis Area and Severity Index.

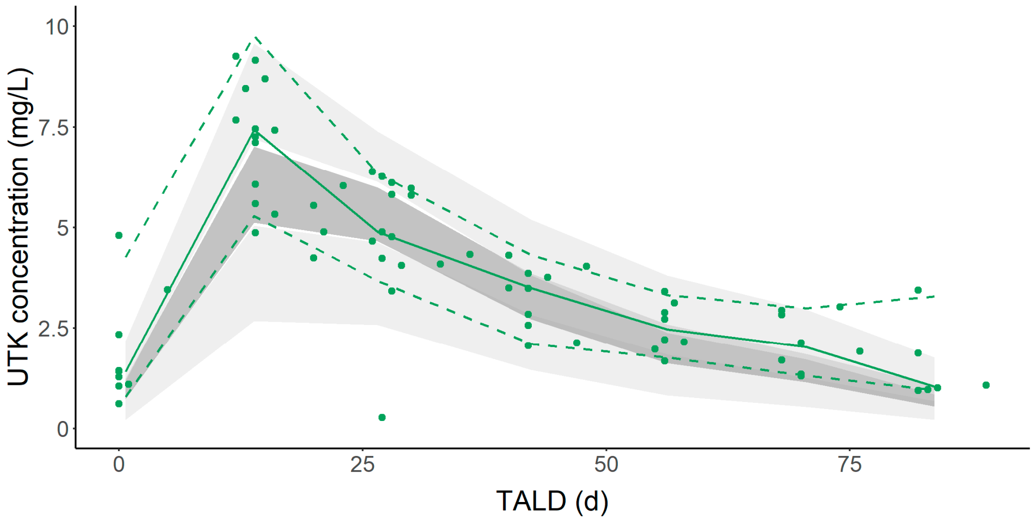

Figure S1. Prediction-corrected visual predictive check obtained from one thousand simulated studies using the selected population PK model. Points represent the observed serum UTK concentrations; lines, 2.5th, 50th, and 97.5th percentiles of the simulated data. Shaded areas represent the 95% prediction intervals of the 5th, 50th, and 95th percentiles of the simulated studies. TALD: time after last dose.

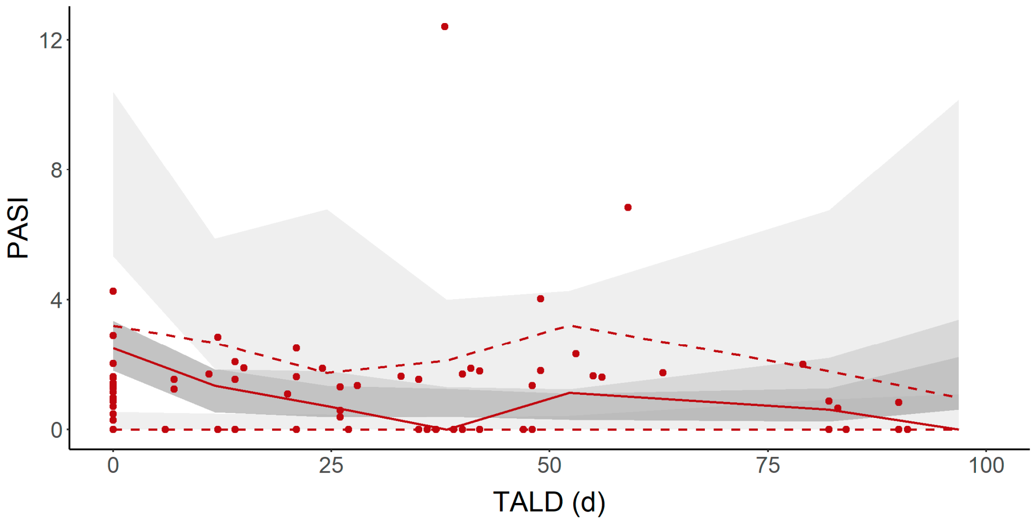

Figure S2. Prediction-corrected visual predictive check obtained from one thousand simulated studies using the selected population PK/PD model. Points represent the observed serum UTK concentrations; lines, 2.5th, 50th, and 97.5th percentiles of the simulated data. Shaded areas represent the 95% prediction intervals of the 5th, 50th, and 95th percentiles of the simulated studies. TALD: time after last dose.

Table S1. Mean of the individual PK/PD parameters draws from the conditional distribution task in Monolix.

ID	k_a (d^{-1})	F (%)	CL (L/d)	V_2 (L)	Q (L/d)	V_3(L)	k_{out}(d^{-1})	I_{max}	$PASI_i$
1	0.40	93	0.20	3.52	0.20	2.10	0.019	0.968	13.722
2	0.15	65	0.18	2.97	0.16	1.68	0.006	0.968	19.995
3	0.15	80	0.16	2.85	0.16	1.59	0.017	0.968	11.616
4	0.13	81	0.19	3.21	0.18	1.87	0.016	0.968	9.075
5	0.09	80	0.17	2.97	0.16	1.72	0.019	0.968	5.564
6	0.11	55	0.16	2.90	0.16	1.76	0.016	0.968	7.346
7	0.20	86	0.24	3.83	0.22	2.53	0.018	0.968	9.860
10	0.14	75	0.20	3.17	0.18	1.78	0.019	0.968	14.508
11	0.33	51	0.25	3.89	0.23	2.52	0.017	0.968	10.643
12	0.18	61	0.18	3.08	0.17	1.98	0.018	0.968	11.624
13	0.15	79	0.24	3.72	0.21	2.31	0.017	0.968	16.094
14	0.17	74	0.19	3.36	0.19	2.56	0.015	0.968	8.238
15	0.19	70	0.19	3.46	0.20	2.33	0.016	0.968	9.736
16	0.09	83	0.18	3.15	0.18	1.85	0.018	0.968	7.360
18	0.14	65	0.30	4.30	0.25	2.85	0.007	0.968	18.845
19	0.17	74	0.17	3.20	0.18	2.15	0.021	0.968	8.616
20	0.22	97	0.12	3.38	0.19	1.89	0.017	0.968	10.079
21	0.15	51	0.21	3.68	0.21	2.78	0.017	0.968	11.062
22	0.19	75	0.13	3.00	0.17	2.21	0.017	0.968	12.280
24	0.21	68	0.23	3.84	0.22	2.73	0.015	0.968	7.104
25	0.21	82	0.17	3.13	0.17	1.96	0.017	0.968	16.983
26	0.17	57	0.21	3.22	0.18	1.87	0.014	0.968	5.963
27	0.25	70	0.14	2.67	0.14	1.73	0.014	0.968	9.557

k_a: absorption rate constant; F: bioavailability CL: clearance; Q: intercompartmental transfer clearance; V_2: central volume of distribution; V_3: peripheral volume of distribution; k_{out}: first-order remission constant rate of psoriatic skin lesion; I_{max}: maximum inhibition drug effect model; $PASI_i$: estimated baseline levels of PASI response.

Table S2. Standard deviation of the individual PK/PD parameters draws from the conditional distribution task in Monolix.

ID	k_a	F	CL	V_2	Q	V_3	k_{out}	I_{max}	$PASI_i$
1	0.02	0.03	0.01	0.04	0	0.27	0.01	0	0.31
2	0.04	0.05	0.01	0.03	0	0.21	0.00	0	0.44
3	0.01	0.06	0.01	0.03	0	0.21	0.01	0	0.29
4	0.03	0.08	0.02	0.04	0	0.21	0.01	0	0.14
5	0.04	0.06	0.02	0.04	0	0.24	0.01	0	0.19
6	0.03	0.05	0.02	0.03	0	0.24	0.01	0	0.20
7	0.02	0.05	0.01	0.04	0	0.28	0.01	0	0.18
10	0.02	0.06	0.02	0.04	0	0.24	0.01	0	0.23
11	0.05	0.04	0.01	0.05	0	0.26	0.00	0	0.27
12	0.02	0.05	0.01	0.04	0	0.23	0.01	0	0.22
13	0.05	0.10	0.02	0.04	0	0.32	0.01	0	0.20
14	0.01	0.04	0.01	0.04	0	0.29	0.01	0	0.20
15	0.03	0.05	0.02	0.04	0	0.30	0.01	0	0.17
16	0.01	0.07	0.02	0.04	0	0.24	0.01	0	0.37
18	0.04	0.11	0.03	0.05	0	0.38	0.01	0	0.60
19	0.02	0.05	0.01	0.04	0	0.27	0.01	0	0.29
20	0.02	0.02	0.00	0.04	0	0.19	0.01	0	0.18
21	0.02	0.04	0.02	0.04	0	0.35	0.01	0	0.21
22	0.03	0.04	0.01	0.03	0	0.26	0.01	0	0.26
24	0.02	0.05	0.02	0.04	0	0.32	0.01	0	0.20
25	0.03	0.05	0.01	0.04	0	0.25	0.01	0	0.19
26	0.03	0.05	0.02	0.04	0	0.25	0.01	0	0.22
27	0.03	0.04	0.01	0.03	0	0.22	0.01	0	0.27

k_a: absorption rate constant; F: bioavailability CL: clearance; Q: intercompartmental transfer clearance; V_2: central volume of distribution; V_3: peripheral volume of distribution; k_{out}: first-order remission constant rate of psoriatic skin lesion; I_{max}: maximum inhibition drug effect model; $PASI_i$: estimated baseline levels of PASI response.

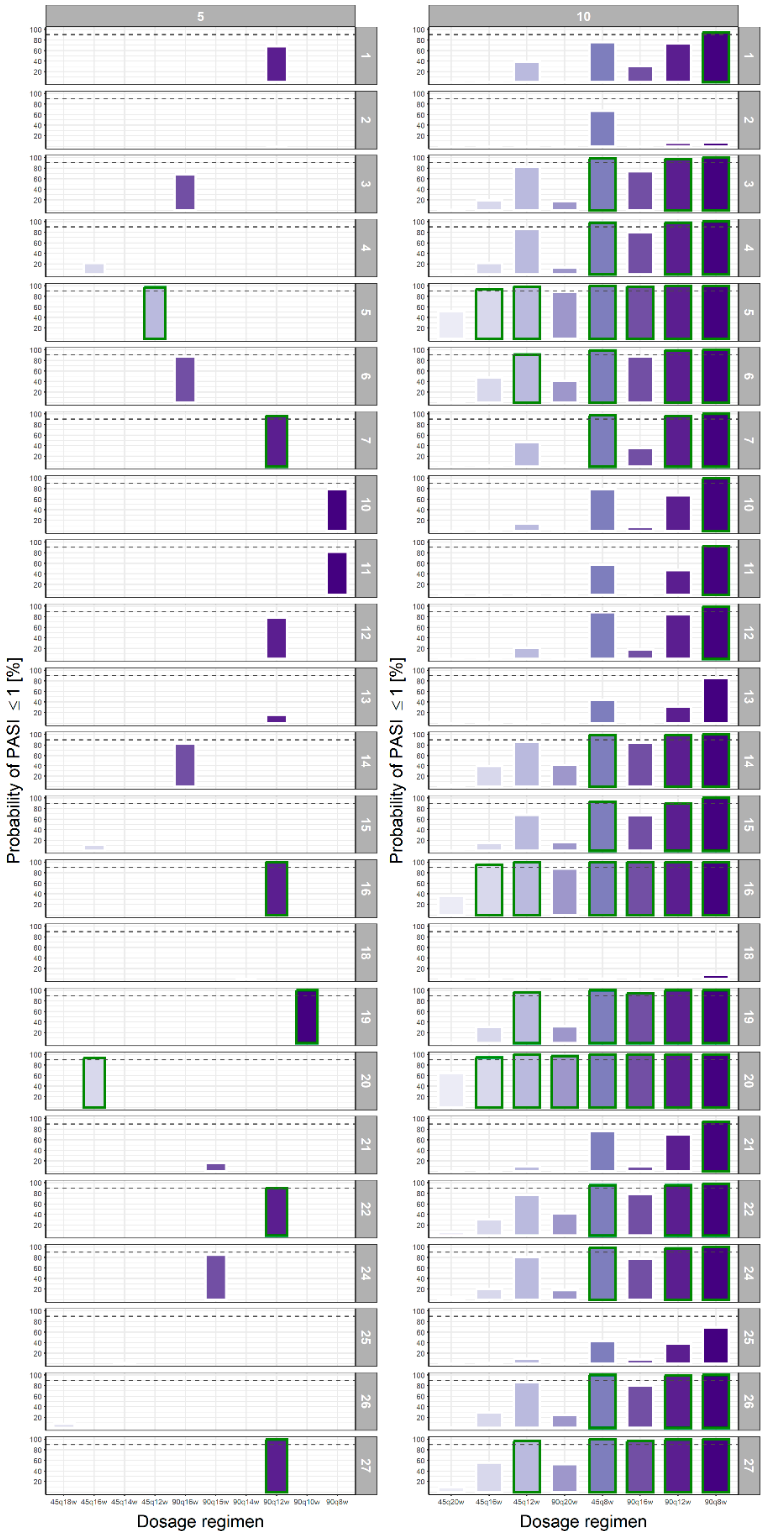

Figure S3. Bar plot of 100 simulated absolute PASI for each patient after UTK administration at cycles 5 and 10, using the individual parameters from the final population PK/PD model and their uncertainties. In green it is represented the probability ≥ 90% of PASI level ≤1. The dose regimens in which a probability ≥ 90% is reached are indicated in green.

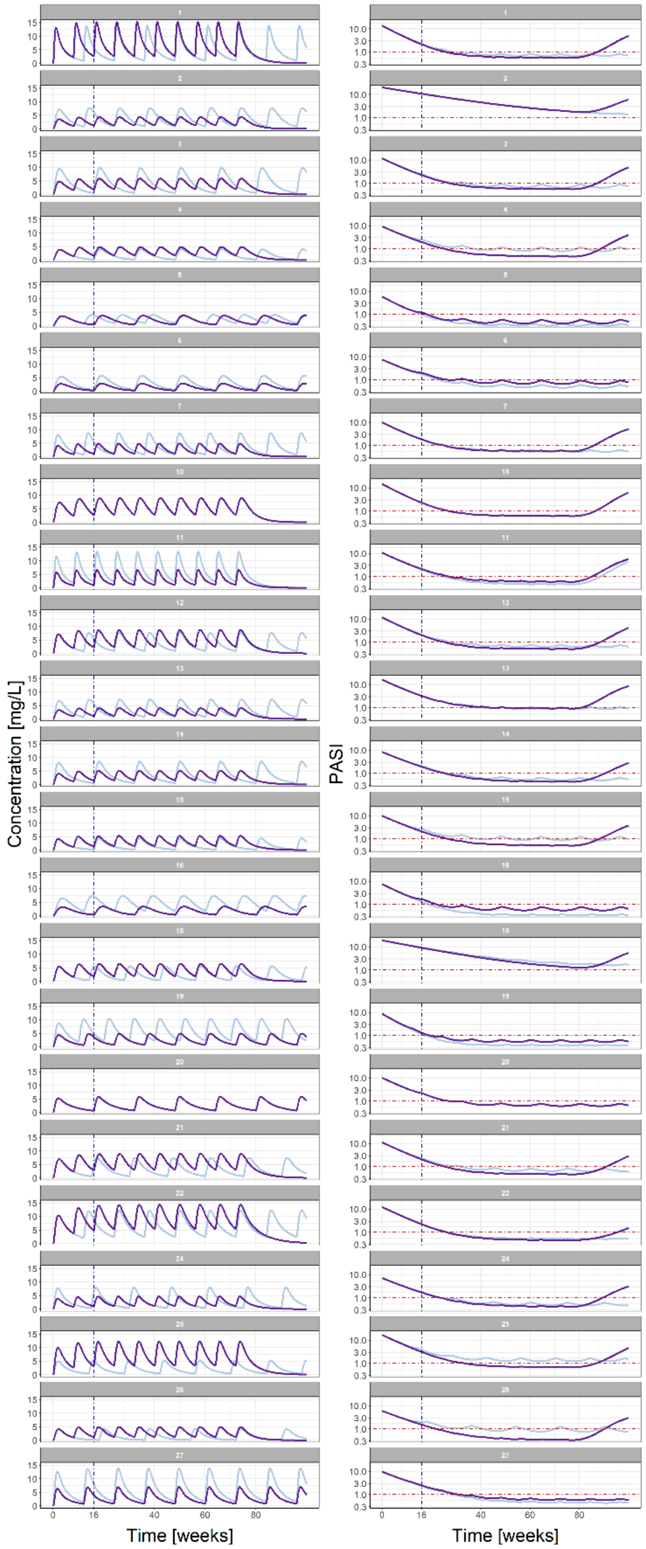

Figure S4. PK and PD simulations with the current dosage regimen from clinical practice (blue) and the individual optimal dosing regimen established after simulations in 10th cycle (purple) for each patient. The vertical dashed line represents the starting point of the maintenance period at week 16. The horizontal dashed line represents the PASI value of 1.
